# Malnutrition among patients with end-stage renal disease in war 2024: the role of healthcare access, dialysis, gender, and economic disparities

**DOI:** 10.1186/s12939-025-02680-3

**Published:** 2025-10-30

**Authors:** Suodad Elhassan, Iyas A. A. Abdelhadi, Nashwa N. S. Mohamed, Aliaa M. A. Mohammed, Waad A. O. Mohammed, Hiba H. M. Abdalla, Amna Khairy

**Affiliations:** 1 Faculty of Medicine, University of Khartoum, Khartoum State, Khartoum, Sudan; 2 Republic of Sudan, Federal Ministry of Health, Field Epidemiology Training Program, Khartoum, Sudan

**Keywords:** Haemodialysis, Healthcare access, End-stage renal disease, Malnutrition, Socio-economic factors

## Abstract

**Background:**

End-stage renal disease patients on haemodialysis are at risk of malnutrition, leading to poor clinical outcomes. This multifaceted issue is expected to worsen due to the ongoing war and displacement crisis. This study aimed to identify the prevalence and factors that contribute to malnutrition among these patients in northern Sudan.

**Methods:**

This cross-sectional study recruited 141 patients from four public haemodialysis centres in the Northern State by cluster sampling. Socioeconomic and clinical data were collected through interviews. Nutritional status was assessed using Subjective Global Assessment (SGA), while healthcare access was evaluated based on Penchansky and Thomas model. Descriptive, bivariate and multivariate analyses were performed using the Statistical Package for Social Science (SPSS) v21, with the statistical significance set at p-value < 0.05.

**Results:**

Most of the patients were males (68.1%), and the mean age was 52 ± 14.5 years. Mild to moderate malnutrition was observed in 44% of the patients, and severe malnutrition in 4.3%. Female gender (OR = 2.29, 95% CI = 1.04–5.07) and dialysis duration over 5 years (OR = 2.95, 95% CI = 1.16–7.51) predicted malnutrition. Better healthcare access was associated with better nutritional status (p-value < 0.05). Lower income, urban residence, hypertension, and cardiopathy were linked to worse nutritional scores.

**Conclusion:**

The study reveals a high prevalence of malnutrition in haemodialysis patients, with a significant contribution of iatrogenic factors, symptomatic burden, healthcare access, and socio-economic disparities. We recommend an integrated healthcare approach that addresses regular nutritional monitoring, symptom management, and socio-economic support.

## Introduction

Chronic Kidney Disease (CKD) is a general term involving heterogenous disorders affecting the structure and function of the kidney, caused by renal injury or a decline in kidney function (Glomerular Filtration Rate [GFR]) lasting three months or more. CKD is classified into five stages according to the GFR, from normal kidney function (stage 1), to end-stage renal disease (ESRD) (stage 5), which requires renal replacement therapy [1, 2]. This ailment is expected to affect more than 10% of the world’s population and is projected to become the fifth leading cause of death by 2040 [3]. Diabetes Mellitus (DM), hypertension (HTN) and cardiovascular disease (CVD) have been recognised as the most prominent reasons leading to CKD [3]. CKD brings about detrimental clinical outcomes, including cardiovascular events, kidney failure necessitating renal replacement treatment, death, and poor quality of life for survivors. Lower levels of kidney function are more inclined to experience complications such as cardiovascular disease (hypertension and uremic cardiomyopathy), anaemia, mineral bone disorder, myopathy, volume overload, electrolytes, and acid-base disturbances [4]. The aforementioned complications can be characterised and quantified, however, complications with ambiguous causes, such as anorexia, fatigue, cachexia, pruritus, nausea, and sexual dysfunction, can cause complex symptoms. Yet, CKD has not been included in the major chronic disease control strategies at a global, regional, and/or national levels despite such disease having an indirect global impact since according to the Global Burden of Disease (GBD) 2015 report; lower glomerular filtration rates were responsible for 1.2 million deaths, 19 million disability-adjusted life-years (DALYs), and 18 million years of life lost due to cardiovascular illnesses [4, 5].

The global prevalence of malnutrition faced by this patient group is 28–54%. It occurs through various iatrogenic and non -iatrogenic mechanisms, and not only tied to reduced oral intake. While non-iatrogenic factors arise naturally from factors that accompany the progression of CKD, iatrogenic factors are unintended consequences of dialysis for patients with ESRD [6]. Haemodialysis treatment itself, encompassing nutrient losses, and inflammation, can result in malnutrition. Non-iatrogenic factors such as insufficient food intake, taste changes, reduced appetite, insulin resistance, catabolism and metabolic derangement, uraemia, and psychological factors all contribute to malnutrition [6]. There has been a large increase lately in the prevalence of protein-energy wasting (PEW) in particular [7]. PEW is a state of disorganised catabolism attributed to metabolic and nutritional imbalances in chronic diseases. As a result; patients with chronic kidney disease (CKD), particularly ESRD, experience muscle wasting, sarcopenia, and cachexia, all of which contribute to morbidity [8]. This can hinder daily activities, reduce strength and autonomy, and negatively impact quality of life (QOL) [9]. Therefore, multidisciplinary management of CKD -including nutritional screening – is pivotal to ensure better clinical outcomes.

The Kidney Disease Outcomes Quality Initiative (KDOQI) provides guidelines about periodic nutritional screening & assessment for CKD patients using a variety of tools; Subjective Global Assessment (SGA) was recommended as a validated and reliable tool for nutritional assessment in ESRD (Level 1B recommendation) [10]. The SGA has been proven to be a good assessment tool in CKD patients [11], it also showed the ability to predict poor outcomes related to malnutrition and mortality in outpatients on haemodialysis [12].

It is worth noting that the management of ESRD is costly and has a major impact on patients, their families’ financial capacity, and the healthcare system in general. According to a study previously conducted in Khartoum State, Sudan, it has been documented that ESRD’s average direct cost is 586 315 Sudanese Pounds (SDGs), equalling $26 174.8 Purchasing Power Parity (PPP) per year, with a median of 38 600 SDGs ($1 723.2 PPP) for medical and non-medical expenses [13, 14].

Some patients might have difficulty paying for substantial out-of-pocket costs for ESRD, especially if their income has decreased due to work absences or being internally displaced from Khartoum State due to Sudan’s ongoing conflict. These expenses – beside the food insecurity status precipitated by the war- can put further challenges on families, and push households into poverty or worsen their existing poverty levels; families may have to choose between medical care and basic necessities like food, leading to malnutrition [7, 14]. Food insecurity, quality, and inaccessibility were found to influence the nutritional status of CKD patients and their ability to comply with the nutritional guidelines, and were linked to adverse clinical outcomes [15]. Furthermore, the armed conflict and the large displacement crisis have resulted in devastating effects on renal care in Sudan, and forced over 70% of patients to interrupt treatment; lack of medications, reduced dialysis sessions and overwhelmed treatment centres were prominent concerns even in safe zones that received displaced dialysis patients [16, 17]. This compromised healthcare access is expected to deprive ESRD patients from diagnostic, educational, and therapeutic services, predisposing them to sub-optimal treatment, clinical deterioration, metabolic derangement, and reduced access to nutritional management and screening services, all of which predispose to malnutrition.

This study aims to determine the prevalence of malnutrition, as the burden is unknown, and the contributing factors, including: clinical and dialysis factors, healthcare access, displacement, and other socioeconomic factors among ESRD patients in Northern Sudan.

## Materials and methods

### Study design and settings

This was a cross-sectional study conducted in four public haemodialysis centres in the Northern State of Sudan, namely Karima Hospital, Al Golid, Wadi Halfa, and Argo Haemodialysis Centres. The state hosted more than 400 000 internally displaced people (IDPs) by March 2024 [18].

### Study population

The study included patients with ESRD receiving periodic haemodialysis at the selected four centres with the following eligibility criteria:

**Inclusion criteria**:

- Sudanese adults (aged 18 or above).
- Both inpatients and outpatients attending the haemodialysis sessions.

**Exclusion criteria**:

- Acutely and severely or terminally ill patients, patients with diagnosed mental or psychological illness, patients with communication barriers, and patients with co-morbidities characterised by energy wasting, e.g. malignancy and tuberculosis (TB).

### Sample size and sampling

Due to the limited research in this field and the limited number of patients, cluster sampling was used, and four out of twelve centres were randomly selected to be covered. All patients who fulfilled the eligibility criteria were included in the study. We identified 183 registered patients during the study period. Six were severely ill patients, and three with communication barriers (hearing impairment, down syndrome, and dementia) were excluded. Also, 33 patients didn’t attend the centre or refused to participate. Therefore, we included 77% (*n* = 141) of all patients registered at the four centres.

### Data collection tool(s) and techniques

Data collection process took place from 8th January to 28th January 2024. We obtained the data on patients’ socio-demographic characteristics, clinical profiles, and haemodialysis parameters through interviews with patients, their carers, and their doctors using a structured questionnaire with close-ended questions. We also cross-checked the clinical data with patients’ records.

### Access to healthcare

We assessed access to healthcare through patient interviews using a validated and standardised questionnaire based on the Penchansky and Thomas model (1981) [19], including the original five dimensions (accessibility, availability, acceptability, affordability, and accommodation), in addition to awareness dimension; an additional sixth dimension that was introduced in a later study by Saurman E [20]. The Questionnaire was originally developed and validated by Hoseini-Esfidarjani et al. (2021) [21], and included a total of 31-items. Each dimension is evaluated through multiple closed-ended questions on a five-point Likert scale.

Responses on the perceived access to healthcare scale were scored from 1 (absolutely disagree) to 5 (absolutely agree), and each domain index and total index were obtained, ranging from 1 to 5. Substitution by the variable’s median was used for the missing data points of the respective question. Healthcare access was categorised into low-level healthcare access (1.00 to 2.33), moderate-level healthcare access (2.34 to 3.67), and higher-level healthcare access (3.67 to 5.00).

### Assessment of patients’ nutritional status

We assessed the patient’s nutritional status using the Subjective Global Assessment (SGA), and patients were categorised into three categories: Category A means the patient is well-nourished, category B means the patient is mildly to moderately malnourished, and category C means the patient is severely malnourished [22, 23]. Patients’ records were used to obtain post-dialysis weight at three points of time: at the time of data collection, retrospectively two weeks before the data collection, and one month before the data collection. Six months of weight change was used when the weight at one month before data collection was missing. Weight loss was scored accordingly, and one point was added in case of weight loss in the past 2 weeks. Patients were interviewed about their food intake, symptoms affecting their oral intake, physical functioning, and diseases and conditions with increased metabolic demand, including fever and corticosteroid usage and doses. Physical observations were collected by evaluating muscle deficit, fat deficit, and oedema in multiple body sites [22]. For each aspect, each site was scored from 0 (no deficit) to 3+ (severe deficit). The point score of the physical exam was determined by the overall subjective rating of the total body deficit, with muscular loss taking precedence over the other two body aspects. The complete PG-SGA score was calculated for all patients according to the scoring sheet/system and were used to determine the appropriate intervention for each patient on the following basis: scores of 0–1 require no intervention, 2–3 scores indicated a need for patient and family education by a professional, with or without a pharmacological intervention according to symptoms survey or other lab results as appropriate. Patients scored 4–8 required dietitian and a physician or nurse intervention. Patients with scores of ≥ 9 critically need symptomatic management and/or nutritional interventions, as appropriate.

All interviews physical examinations and assessments were conducted by trained health professionals; all are proficient speakers of the patients’ local language. Weight scales were calibrated and patients were weighted based on the standard operating procedures of the centre.

We piloted the study tools by interviewing 20 patients from the same study settings to assess the face validity and refine the questionnaire. Patients feedback about the relevance and clarity of questions was gathered. The questionnaire was modified accordingly, and more appropriate language was adopted. The sequence of the questions was revised and edited, and 4 redundant questions were deleted.

### Data management and analysis

We coded, entered, and analysed data using the Statistical Package for Social Science (SPSS)^®^ version No. 21 software for statistical analysis.

Descriptive statistics were used to present demographic and socioeconomic profiles, estimate the prevalence of each nutritional category, and describe the clinical profile of the patients and symptoms affecting oral intake.

We performed independent sample T-test and One-way analysis of variance (ANOVA) to detect differences in SGA score between different categories. Independent Chi-square test and Fisher’s exact test were used to examine differences in the nutritional status according to the demographic, socio-economic, and clinical variables.

Multivariable logistic regression was used to identify the predictors of malnutrition at a 95% confidence level. Statistical significance was set at a level of *p* < 0.05.

### Ethical considerations

All methods were performed in accordance with the ethical principles outlined in the Declaration of Helsinki. Ethical clearance was issued from the ethics committee of the Northern State Ministry of Health, Sudan. Informed consent was taken from all participants to fill the questionnaire and to conduct the nutritional assessment. Participants had the right to withdraw at any time without consequences. Data were kept confidential. Permission from facilities administrations was obtained, and no obstruction of healthcare delivery or workflow occurred.

## Results

### Demographic and socioeconomic characteristics of study participants

The study included 141 ESRD patients recruited from four haemodialysis centres in the Northern State in Sudan as follows: 54 patients (38.3%) from Karima centre, 44 (31.2%) from Algolid, 24 (17%), and 19 (13.5%) from Argo, and Wadi Halfa, respectively. Patients ages ranged between 19 and 82 years, with a mean of 52 years, and 68.1% of them were males.

Half of the patients suffered displacement since the start of the war at their original residence, while almost half of them lived with their relatives/ extended family houses (55.3%), about 2% lived in displacement camps or settlements. The majority of patients had a relatively low income of less than 150 k SDG per month (56.7%) (Table 1).

**Table 1:** Sociodemographic and clinical characteristics of the ESRD on haemodialysis at Northern Sudan. *N* = 141

Sociodemographic and clinical characteristics of the Patients		*N* (%)
Treating Centre	Karima Teaching Hospital	54 (38.3)
	Algolid Dialysis Centre	44 (31.2)
	Argo Dialysis Centre	24 (17.0)
	Wadi Halfa Dialysis Centre	19 (13.5)
Age	< 45	44 (31.2)
	45–65	66 (46.8)
	>= 65	31 (22.0)
	M(SD)	52 (14.5)
Gender	Male	96 (68.1)
	Female	45 (31.9)
Marital Status	Single	22 (15.6)
	Married	105 (74.5)
	Divorced or widowed	13 (9.2)
	Missing	1 (0.7)
Education	illiterate	12 (8.5)
	Primary	49 (34.8)
	Secondary	48 (34.0)
	University or higher	31 (22.0)
	Missing	1 (0.7)
Occupation	Retired or non-working	111 (78.7)
	Free worker	19 (13.5)
	Formal Employee	10 (7.1)
	Missing	1 (0.7)
Income/ Month in SDG	Less than 150 K	80 (56.7)
	150–300	41 (29.1)
	Above 300 K	18 (12.8)
	Missing	2 (1.4)
Displacement	Yes	70 (50.0)
	No	70 (50.0)
	Missing	1 (0.7)
Household	Own house	48 (34.0)
	Rented house	11 (7.8)
	Relative/extended family	78 (55.3)
	IDP camp	3 (2.1)
	Missing	1 (0.7)
Residence	Rural	89 (63.1)
	Urban	47 (33.3)
	Missing	5 (3.5)
Comorbidities	None	23 (16.3)
	HTN	104 (73.8)
	Diabetes	16 (11.3)
	Cardiac diseases	12 (6.1)
	Liver diseases	2 (1.4)
	Hyperuricemia and Gout	8 (5.7)
	Neurological diseases	9 (6.4)
	Other	14 (9.9)
Hospitalisation status	Yes	6 (4.3)
	No	135 (95.7)
Recent Hospitalisation history	Yes	37 (26.2)
	No	104 (73.8)
ESDR Aetiology	Hereditary and Congenital	29 (20.6)
	Diabetic Nephropathy	10 (7.1)
	Obstructive Uropathy	10 (7.1)
	HTN	52 (36.9)
	Uric acid nephropathy	7 (5.0)
	Autoimmune	4 (2.8)
	Pyelonephritis	3 (1.8)
	Systematic infections (Malaria, typhoid)	2 (1.4)
	snake envenomation, medications toxicity	2 (1.4)
	I don’t know	24 (17.0)
	Other (unidentified)	21 (14.9)
Haemodialysis Frequency	Once/ week	3 (2.1)
	Twice/ week	134 (95.0)
	3 or more/ week	3 (2.1)
	Missing	1 (0.7)
Haemodialysis Adequacy	Yes	128 (90.8)
	No	12 (8.5)
	Missing	1 (0.7)
Haemodialysis Duration	Less than 2 years	39 (27.7)
	2–5 years	44 (31.2)
	More than 5 years	58 (41.1)
	Mean (SD)	4.6 (4.7)
Haemodialysis Regularly	Yes	136 (96.5)
	No	5 (3.5)
Adherence to Nutritional/ diet Plans	No plan provided	25 (17.7)
	Not adherent	42 (29.8)
	Adherent	74 (52.5)
Malnutrition	Well- Nourished (A)	73 (51.8)
	Mildly to Moderately Malnourished (B)	62 (44.0)
	Severely Malnourished (C)	6 (4.3)
	Mean score (SD)	11.1 (6.5)
Health care access	Low Level	0 (0)
	Moderate Level	54 (38.3)
	High Level	83 (58.9)
	Missing	4 (2.8)
	Mean Access Index (SD) =	3.9 (0.5)

### Clinical profile of study participants

Hypertensive renal damage was the most frequent predisposing aetiology to ESRD (identified in 36.9% of patients), followed by hereditary and congenital kidney diseases (20.6%) (including Polycystic Kidney Disease), diabetic nephropathy and obstructive uropathy (7.1%). Other identified aetiologies were autoimmune diseases (2.8%), medication-induced and snake envenomation-induced kidney injury (1.4%), and systemic infections (Malaria and Typhoid fever) (1.4%). The rest of the patients reported unknown or miscellaneous causes (31.9%). About 83.7% of ESRD patients had chronic comorbidities. Hypertension (HTN) was the most prevalent condition, affecting 73.8% of patients, followed by diabetes (11.3%) and cardiopathy (8.5%) (Table 1).

Six patients (4.3%) had been hospitalised during the data collection period, mainly due to electrolyte disturbances, hypertensive crisis, or severe hyperuricemia, while 37 (26.2%) had a history of hospitalisation during the last six months, predominantly due to electrolytes disturbances (29.6%). Regrading dialysis parameters, patients were on dialysis for as long as 30 years; with a mean of 4.6 years.

### Prevalence of malnutrition and dietary plans

About half of the patients (51.8%) were categorised as well-nourished (category A), 44% were mildly to moderately malnourished (category B), and 4.3% were severely malnourished (category C).

According to the nutritional triage recommendations based on the PG-SGA point score, only 4 patients (2.8%) required no intervention at that time (scored 0–1), 14 patients (9.9%) needed patient and family education by a by dietitian, nurse, or other clinician with pharmacologic intervention (as appropriate), and 40 patients (28.4%) required an intervention by a dietician, in conjunction with nurse or physician as indicated by symptoms. Moreover, more than half of the patients (58.9%) had a critical need for improved symptom management and/or nutritional intervention (scored ≥ 9). However, only about the half (52.5%) had a dietary plan they were adherent to, the rest of the patients either didn’t have any (17.7%) or were not adherent (29.8%) (Table 1).

The symptomatic contribution to the overall SGA score was as high as 16, with a mean of 5.6 ± 4.9. Only 22.7% were free of any symptom affecting their oral intake, while 77.3% had at least one symptom minimising intake. Feeling full quickly (52.5%), fatigue (39.7%), lack of appetite (39.7%), and dry mouth (34.0%) were the most frequently reported symptoms. There was a significant difference between males and females regarding perceived intake adequacy, 83.3% of males reported adequate intake, in contrast to only 57.8% of females (p-value = 0.002).

### Bivariate analysis for the factors associated with malnutrition

The factors associated with malnutrition were tested through bivariate analysis. These were the demographic, socioeconomic, nutritional, clinical factors, and access to health care.

**Fig. 1:**
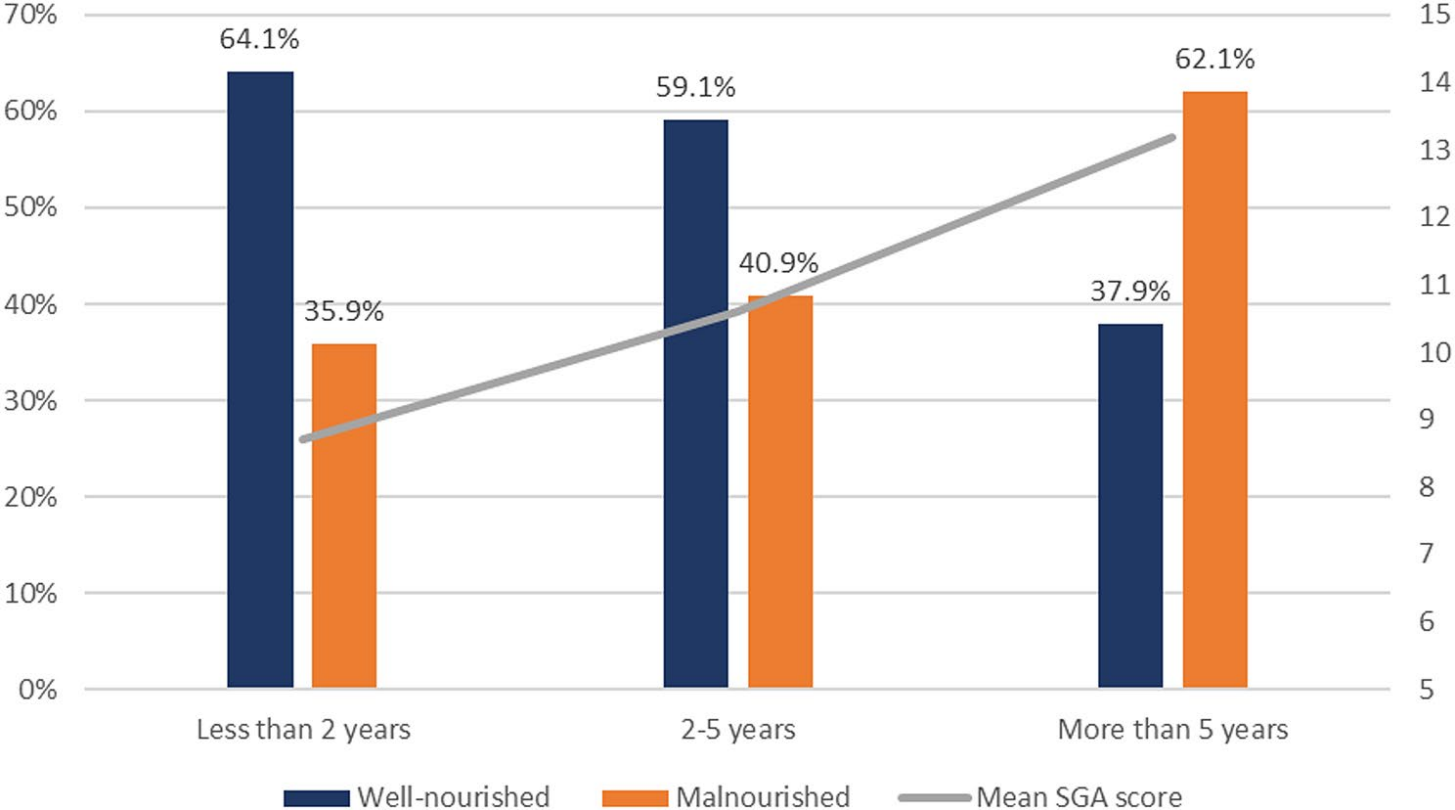
Malnutrition in Relation to Dialysis Duration among Patients with ESRD, *n* = 141

Among the four haemodialysis parameters measured, dialysis duration was the only factor impacting the nutritional status; 64.1% of patients started haemodialysis in less than two years were well-nourished (category A), compared to only 37.9% of patients on haemodialysis for more than five years (p-value = 0.021) (Fig. 1) (Table 2).

**Table 2:** Bivariate analysis for the clinical and socioeconomic determinants of malnutrition among patients with ESRD on haemodialysis at Northern Sudan 2024. *N* = 141

		Malnutrition		P Value	Mean (Sd)	P-value
		No	Yes			
		N (%)	N (%)			
Age	< 45	22 (50.0)	22 (50.0)	0.818	10.9 (6.8)	0.501
	45–65	36 (54.5)	30 (45.5)			
	>= 65	15 (48.4)	16 (51.6)		12.3 (7.3)	
Gender	Male	55 (57.3)	41 (42.7)	0.083	10.9 (6.5)	0.445
	Female	18 (40.0)	27 (60.0)		11.8 (6.6)	
Education	illiterate	3 (25.0)	9 (75.0)	0.331	13.3 (8.8)	0.405
	Primary	26 (53.1)	23 (46.9)		11.8 (6.5)	
	Secondary	26 (54.2)	22 (45.8)		9.7 (5.6)	
	University	15 (55.6)	12 (44.4)		11.6 (7.2)	
	Higher education	3 (75.0)	1 (25.0)		12.0 (3.4)	
Occupation	Retired/ non-working	56 (50.5)	55 (49.5)	0.731	11.0 (6.6)	0.467
	Free worker	11 (57.9)	8 (42.1)		11.0 (6.8)	
	Formally employed	6 (60.0)	4 (40.0)		13.6 (4.6)	
Income per month	Less than 150k	37 (46.3)	43 (53.8)	0.194	13.3 (6.1)	< 0.001**
	150–300 k	26 (63.4)	15 (36.6)		8.7 (5.7)	
	More than 300k	10 (55.6)	8(44.4)		7.8 (7.0)	
Displacement	Yes	36 (51.6)	34 (48.6)	1.000	12.0 (6.6)	0.140
	No	37 (52.9)	33 (47.1)		10.4 (6.4)	
Household	Relative/extended family	40 (51.3)	38(48.7)	0.133	12.7 (6.1)	0.005*
	Own house	29 (60.4)	19 (39.6)		7.8(5.3)	
	Rented house	4 (36.4)	7 (63.6)		13.5 (9.1)	
	IDP camp	0 (0.0)	3 (100.0)		16.3 (3.4)	
Residence	Urban	23 (48.9)	24 (51.1)	0.901	13.6 (6.7)	0.001*
	Rural	46 (51.7)	43 (48.3)		9.8(6.1)	
Comorbid conditions presence	Yes	59 (50.0)	59 (50.0)	0.468	11.8 (6.4)	0.012*
	No	14 (60.9)	9 (39.1)		8.0 (6.2)	
Comorbidities/ Diabetes	Yes	8 (50.0)	8 (50.0)	1.000	13.9 (7.7)	0.068
	No	65 (52.0)	60 (48.0)		10.8 (6.3)	
Comorbidities/ HTN	Yes	50 (48.1)	54 (51.9)	0.200	12.3 (6.4)	< 0.001**
	No	23 (62.2)	14 (37.8)		8.0 (5.7)	
Comorbidities/Cardiac	Yes	6 (50.0)	6 (50.0)	1.000	15.3 (7.1)	0.019*
	No	67 (51.9)	62 (48.1)		10.8 (6.3)	
Hospitalisation	Yes	3 (50.0)	3 (50.0)	1.000	13.3 (4.0)	0.401
	No	70 (51.9)	65 (48.1)		11.0 (6.6)	
Recent Hospitalisation History (within 6 m)	Yes	17 (45.9)	20 (54.1)	0.526	12.6 (6.4)	0.114
	No	56 (53.8)	48 (46.2)		10.6 (6.5)	
Haemodialysis Frequency/ week	Once	2 (66.7)	1 (33.3)	1.000	11 (13.1)	0.598
	Twice	69 (51.5)	65 (48.5)		11.2 (6.4)	
	3 or more	2 (66.7)	1 (33.3)		5.0 (2.0)	
Haemodialysis Adequacy	Yes	65(50.8)	63 (49.2)	0.453	11.0 (6.3)	0.619
	No	8 (66.7)	4 (33.3)		12.3 (8.3)	
Haemodialysis Duration	Less than 2 years	25 (64.1)	14 (35.9)	0.021*	8.7 (5.8)	0.003*
	2–5 years	26 (59.1)	18 (40.9)		10.6 (6.7)	
	More than 5 years	22 (37.9)	36 (62.1)		13.2 (6.3)	
Haemodialysis Regularly	Yes	70 (51.5)	66 (48.5)	1.000	11.1 (6.4)	0.611
	No	3 (60.0)	2 (40.0)		12.6 (9.3)	
Adherence to Nutritional Plans	No plan provided or non-adherent	37 (55.2%)	30 (44.8%)	0.435	11.0 (6.2)	0.847
	Adherent	36 (48.6%)	38 (51.4%)		11.2 (6.8)	
Healthcare access index	Moderate level Access	22 (40.7)	32 (59.3)	0.036*	13.7 (5.7)	< 0.001**
	High level access	49 (59.0)	34 (41.0)		9.7 (6.5)	

* Association is significant at the level of 0.05

** Association is significant at the level of 0.001

**Fig. 2:**
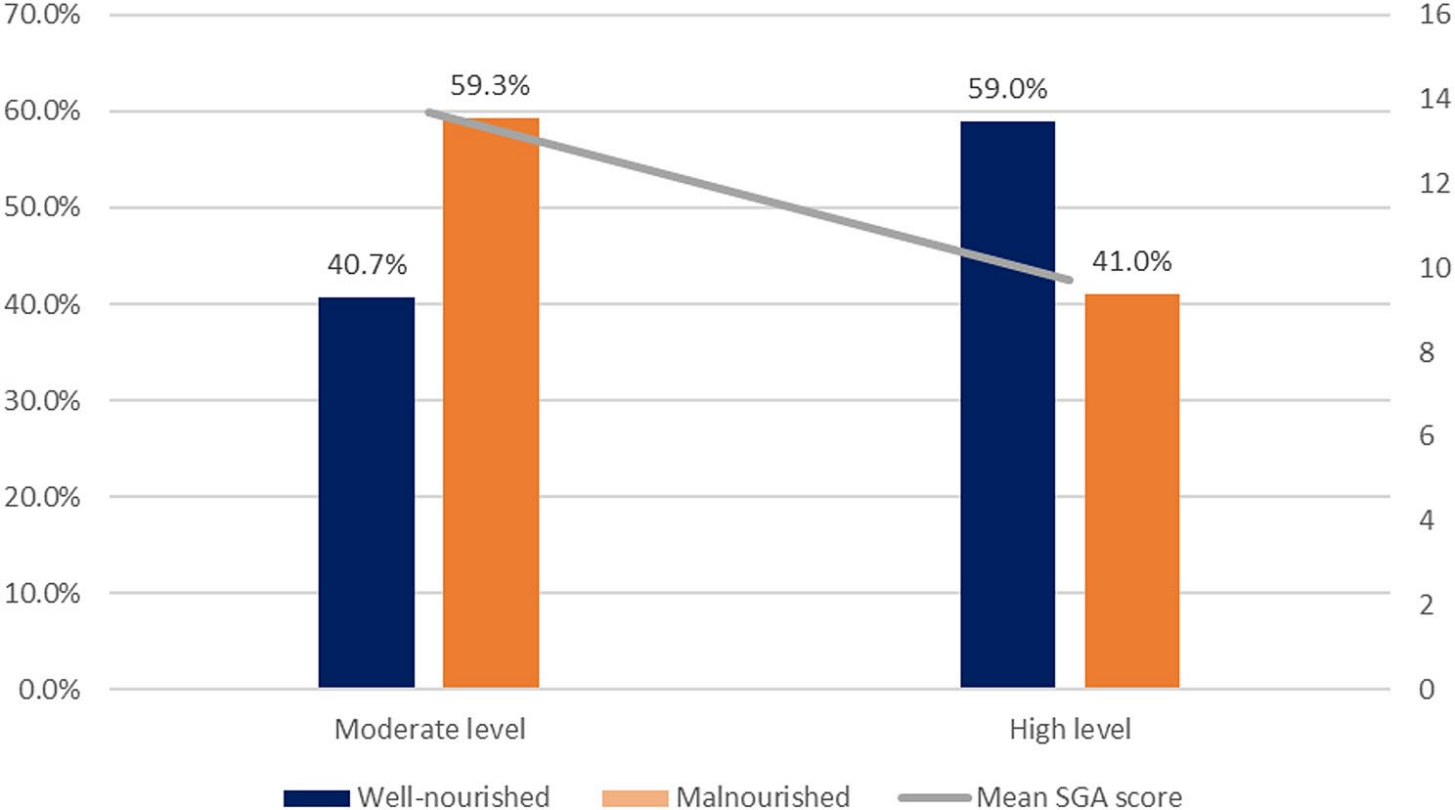
Malnutrition Prevalence in each Healthcare Access Level among Patients with ESRD, *n* = 141

Patients with the higher level of healthcare access had significantly lower rates of malnutrition compared to patients with the moderate level (p-value = 0.036) (Fig. 2), and had lower SGA scores by an average of four points (Table 2).

The demographics, socio-economic, and other clinical parameters were not significantly associated with being malnourished; however, the lower income level, urban residence, and house non-ownership were associated with a significantly higher SGA score, as well as having comorbid HTN or cardiac disease (Table 2).

### Predictors of malnutrition using binary logistic regression

The binary logistic regression model contained five variables (gender, age, income, dialysis duration, and healthcare access) (Table 3).

The strongest significant predictor was the haemodialysis duration; the odds of developing malnutrition were significantly higher among patients undergoing dialysis for more than 5 years, compared to patients of less than two years of dialysis, with an adjusted odds ratio of 2.95 (95% CI = 1.16–7.51, p-value = 0.024).

Gender was also a significant predictor; the odds of having malnutrition were 2.3 times higher in females compared to males (AOR = 2.29, 95% CI = 1.04–5.07, p-value = 0.040).

Age, income, and healthcare access showed no significant contribution in predicting malnutrition in this model (P-value ˃ 0.05) (Table 3).

**Table 3:** Multi- variable logistic regression analysis for the predictors of malnutrition among patients with ESRD on haemodialysis at Northern Sudan 2024. *N* = 141

Variable	B	S.E.	*P*-value	Odd Ratio	OR (95% CI)	
**Gender** (ref: Male)	0.829	0.405	0.040*	2.292	1.037	5.066
**Age** (ref: Less than 45 years)			0.451			
45–65 Years	-0.385	0.431	0.372	0.680	0.292	1.585
More than 65 Years	0.199	0.551	0.718	1.220	0.414	3.592
**Income/month (ref: Less than 150 K)**	-0.271	0.418	0.517	0.763	0.336	1.732
**Haemodialysis Duration** (ref: less than 2 years)			0.052			
2–5 years	0.285	0.488	0.559	1.330	0.551	3.458
More than 5 years	1.080	0.447	0.024*	2.945	1.155	7.505
**Healthcare access** (ref: Moderate level)	-0.705	0.413	0.088	0.494	0.220	1.110
*Classification percentage correct*,* 64.7%; Cox & Snell R square*,* 0.123; Nagelkerke R square*,* 0.164; Omnibus test*,* 0.012; Hosmer & Lemeshow; 0.761*,* AUC*,* 70.4%*						

* Significant at the level of 0.05

## Discussion

Malnutrition is a serious concern among patients with end-stage renal disease (ESRD) and significantly affects morbidity, mortality, quality of life, and clinical outcomes. This study explores the potential multi-faceted nature of malnutrition in ESRD, including the potential overlap with the patients’ clinical characteristics, the iatrogenic contribution, and the socio-economic profile, especially in the Sudan war context and the consequent displacement crisis and healthcare disruption.

The study reports an alarmingly high rate of malnutrition affecting almost half of patients with ESRDs in war time. This is quite similar to the findings of a single-centre study conducted in Palestine where 47.2% were malnourished [24]. In a second study in Palestine, the rate was estimated to be 45.4% [25]. Malnutrition was found in 48.3% of haemodialysis patients in our study, with 44% having mild-to-moderate malnutrition and 4.3% suffering from severe malnutrition. According to a Jordanian study that examined the prevalence of malnutrition among patients with ESRD, about 62% suffered from malnutrition [26]. Similarly, a study conducted in Iraq (*n* = 86) concluded that 63.5% of patients had malnutrition (moderate, 45.9%; severe, 17.6%) [27]. Thus, despite the high rate of malnutrition, it was neither bizarre nor particularly unusual when compared to the ESRD populations in other countries that suffered conflicts. Other countries have shown lower rates of malnutrition- using the same assessment tool - such as Australia (20%) [11], and Morocco (29%) [28]. Discrepancies in socioeconomic, environmental, and dietary factors and treatment approaches may account for the variations in the prevalence of malnutrition.

Being on haemodialysis for more than five years was a direct predictor of malnutrition. This points to the significant iatrogenic contribution to its pathophysiology in this population. This result is consistent with another study in Vietnam [29]. It was previously stated that the dialysis procedure may potentiate Protein Energy Wasting (PEW), possibly through loss of protein into dialysate, induced catabolism, and concurrent symptoms [30]. In countries with limited resources, reuse of the dialyzer -which is a common practice- carries a risk for infections, as well as immunogenic and biochemical reactions, and increased membrane permeability, eventually leading to inflammation and nutrient loss [6]. A cohort study found that significantly higher CRP levels were present in malnourished ESRD patients [31]. Other studies confirmed a strong relationship between SGA B & C categories and high CRP [32], hence, pointing to what so called: “malnutrition-inflammation complex syndrome”, characterized by hypercatabolic state and low appetite [33].

In our study, female gender was a significant predictor for malnutrition among ESRD patients; they had 2.3 higher odds of having malnutrition compared to males (CI = 1.037–5.066). This result is consistent with the findings of previous studies [29, 34]. This hypothesis could be justified by physiological differences between males and females in terms of unique nutritional requirements, for example, in pregnancy and lactation. Also, this finding could be linked to the gender inequality in access to quality diet; a situation that was worsened by the conflict. By September 2024, UN has reported 84% of women in Sudan are unable to meet the minimum acceptable diet [35]. It is worth mentioning that a cohort study concluded that SGA predicted poor outcomes in both sexes, while other methods (serum albumin, hand grip strength) were important independent predictors in males only [31]. Potentially greater psychological challenges in females may directly interfere with appetite and food intake [36]; in our study, females with ESRD had significantly lower dietary intake than males.

While the rest of socio-economic factors were not significant impactors of the final classification of patients, many of them correlated significantly with higher SGA scores, indicating a tendency or a potential toward malnutrition (in addition to the need of more advanced interventions), such as lower income levels; potentially leading to a lesser access to nutrients packed with lean protein. A similar finding was reported in previous research articles [37, 38]. In this study, house ownership emerged as a socioeconomic indicator of malnutrition. This is an essential parameter for the Sudan context and war, as many displaced families tend to spend a substantial part of their household income on rent, leading to financial hardship and affecting their ability to obtain basic needs, such as providing proper nutrition for the family. This specifically increases vulnerability and can be further supported by the fact that lower income levels had significantly higher scores; due to their greater control over housing expenses and their ability to spend more on food, homeowners are usually more likely to be food secure than renters. Furthermore, owning a home can provide a sense of security and stability, both of which can improve health outcomes [39, 40]. This, in turn, can explain why urban residence was linked to higher scores in terms of increased rent and life expenses. Although a more comprehensive study is required to validate this connection. Our study aids in formulating a hypothesis between the two entities.

Notably, the SGA scores of our patients were very high considering the overall malnutrition rate. However, we believe that in our population, the symptomatic burden could have substantially contributed to this increase; less than one-fourth were free of symptoms restricting their intake. One of the perks of the SGA tool is that it utilizes many factors- such as intake, daily functioning, and weight stability- to diagnose nutritional deficit, rather than relying traditionally on wasting signs and low Body Mass Index (BMI); in fact, physical wasting contribution to the final score is relatively low (3 points); and on reverse, many patients classified in category (A) have some degree of muscle deficit with recent clinical or functional improvement and normal intake, and sometimes weight gain (suggesting cachexia or a chronic deficit but with recent stabilization of weight and intake, which is expected in such a patient subset) [41]. Malnutrition diagnosis using SGA considered weight stability, and the common- but stable- muscle wasting in CKD patients.

Patients with comorbid HTN or cardiopathy had higher scores, potentially due to poorer general condition, inflammatory process, metabolic alteration, cardiac cachexia [30], and GI symptoms exacerbated by medication [42]. The complex interaction of comorbidities and medication side effects can create a vicious cycle in which malnutrition worsens the underlying disease and increases the risk of further complications. This is supported by the fact that higher healthcare access had less incidences of malnutrition, indicating a role for appropriate renal care and possibly comorbidity control to mitigate malnutrition risk. Comorbidities increase the risk of malnutrition in ESRD and overall poor clinical outcomes in malnourished subjects [43]. This can be important in the context of war; given that half of our sample is displaced, they may suffer nutritional and healthcare restrictions in conflict zones and in their displacement journey.

The present study has several strengths; first, it provides a feasible, valid, and comprehensive assessment of nutrition rather than adopting a screening approach. The Kidney Disease Quality Outcome Initiative (KDQOI) recommended the SGA as a reliable method to be used in chronic kidney disease (level 1B recommendation) [10]. Importantly, this study offers insights into the levels of dietary and clinical interventions required. One of the possible limitations of this study is recall bias; given that some data were collected retrospectively. Poor documentation and under-availability of documented laboratory markers were barriers to using these data in complementation with the SGA. The cross-sectional design prevents causal inferences, and the observational nature of the study means that residual confounding factors cannot be ruled out. Future longitudinal research is recommended to investigate iatrogenic causality- including medications- of malnutrition in ESRD in the context of the under-resourced health system in Sudan, and the associated clinical outcomes and healthcare disparities.

## Conclusion

This research explores the issue of malnutrition among end-stage renal disease (ESRD) patients, particularly within the context of the Sudanese armed conflict, the displacement crisis, and anticipated limitations in access to optimum care.

A relatively high rate of malnutrition was found in this population that was closely associated with the socioeconomic profile, dialysis duration, and access to healthcare. The study indicates that female gender and long periods of dialysis are significant predictors of malnutrition, pointing up to the importance of addressing the iatrogenic contribution of haemodialysis in malnutrition in this patient’s subset in future protocols. Socioeconomic stress, especially housing and income instability due to war, further aggravate the issue, necessitating an integrated, multidisciplinary clinical approach, focusing on continuous nutritional monitoring, proper symptom management, and equity in access to care. Longitudinal studies are recommended for future research to provide more evidence on the long-term iatrogenic influence, including medications.

## Data Availability

The data of this study are not publicly available due to ongoing secondary analysis and research work. Access to the dataset is therefore restrcited to perceive integrity of the currenct investigations. However, data are available from the corresponding author upon reasonable request.

## Abbreviations

ANOVA: Analysis of Variance
AOR: Adjusted Odd Ratio
BMI: Body Mass Index
CI: Confidence Interval
CKD: Chronic Kidney Disease
CRP: C-Reactive Protein
CVD: Cardiovascular Disease
DALYs: disability-adjusted life-years
DM: Diabetes Mellitus
ESRD: End-Stage Renal Disease
GBD: Global Burden of Disease
GFR: Glomerular Filtration Rate
GI: Gastro Intestinal
HIV: Human Immunodeficiency Virus
HTN: Hypertension
IDP: Internally displaced people
KDQOI: Kidney Disease Quality Outcome Initiative
M: Mean
nPNA: normalized protein nitrogen appearance
PEW: Protein Energy Wasting
PPP: Purchasing Power Parity
QOL: Quality of Life
SDG: Sudanese Pound
SD: Standard deviation
SGA: Subjective Global Assessment
SPSS: Statistical Package for Social Science
TB: Tuberculosis
UN: United Nations
